# Current Methods to Investigate Nociception and Pain in Zebrafish

**DOI:** 10.3389/fnins.2021.632634

**Published:** 2021-04-08

**Authors:** Nils Ohnesorge, Céline Heinl, Lars Lewejohann

**Affiliations:** ^1^German Federal Institute for Risk Assessment (BfR), German Centre for the Protection of Laboratory Animals (Bf3R), Berlin, Germany; ^2^Institute of Animal Welfare, Animal Behavior and Laboratory Animal Science, Freie Universität Berlin, Berlin, Germany

**Keywords:** zebrafish, animal model, pain, nociception, algogen, analgesic, neuroimaging, refinement

## Abstract

Pain is an unpleasant, negative emotion and its debilitating effects are complex to manage. Mammalian models have long dominated research on nociception and pain, but there is increasing evidence for comparable processes in fish. The need to improve existing pain models for drug research and the obligation for 3R refinement of fish procedures facilitated the development of numerous new assays of nociception and pain in fish. The zebrafish is already a well-established animal model in many other research areas like toxicity testing, as model for diseases or regeneration and has great potential in pain research, too. Methods of electrophysiology, molecular biology, analysis of reflexive or non-reflexive behavior and fluorescent imaging are routinely applied but it is the combination of these tools what makes the zebrafish model so powerful. Simultaneously, observing complex behavior in free-swimming larvae, as well as their neuronal activity at the cellular level, opens new avenues for pain research. This review aims to supply a toolbox for researchers by summarizing current methods to study nociception and pain in zebrafish. We identify treatments with the best algogenic potential, be it chemical, thermal or electric stimuli and discuss options of analgesia to counter effects of nociception and pain by opioids, non-steroidal anti-inflammatory drugs (NSAIDs) or local anesthetics. In addition, we critically evaluate these practices, identify gaps of knowledge and outline potential future developments.

## Introduction

Pain in fish poses an underappreciated ethical and technical concern in animal experiments (Carbone, 2011; Gaskin and Richard, 2012; Sloman et al., 2019) but it also offers new possibilities for insights and discoveries in pain and drug research. Pain and nociception are evolutionary well-conserved mechanisms with an important role for the survival of animals (Milinkeviciute et al., 2012; Crook et al., 2014) and consequently many *in vivo* and *in vitro* models to study nociception and pain have been developed (Gregory et al., 2013; Wainger et al., 2015; Graham, 2016). In fish, some models still need more fine-tuning where others are already well established. The number of zebrafish used in animal experiments has been continuously rising over the past years and in the European Union is only outnumbered by mice and rats (European Commission, 2020). The advantages of zebrafish as animal models are among others high fecundity and offspring numbers, short generation time, low housing costs and rapid external development of transparent embryos making it accessible for applications like large-scale forward genetics, screenings and imaging techniques.

In respect to pain models, the neural process to detect harmful or potentially harmful stimuli known as nociception has been well studied. Nociceptors are sensory neurons with free nerve endings equipped with a repertoire of receptors capable to detect noxious stimuli. One important family of receptors are polymodal transient receptor potential channels (TRP channels) of which the most prominent members Trpv1, Trpa1a, and Trpa1b are homologs to human TRPV1 and TRPA1, respectively (Prober et al., 2008; Pan et al., 2012; Gau et al., 2013). These receptors respond to a wide variety of stimuli, like changes in temperature, pH or mechanical pressure. Channel activation causes an influx of sodium and calcium and can lead to the creation of an action potential. Depending if the nociceptor is located in the head or the rest of the body, this signal is then conveyed either via the trigeminal ganglia (TG) or via the dorsal root ganglia (DRG) and the dorsal horn to the brain (Caron et al., 2008; Malafoglia et al., 2013).

Whereas nociception is a well-known process in all vertebrates, it has been controversially discussed if fish are capable of experiencing pain (Rose et al., 2014; Sneddon, 2015). The main issue lies in the definition of pain by the International Association for the Study of Pain (IASP) as *“an unpleasant sensory and emotional experience”* and the resulting question, if the fish brain is sufficiently developed to allow for these feelings or if this is an exclusive mammalian trait (IASP, 1979). Mirrored by the discussions for birds, reptiles and cephalopods, there is mounting evidence that suggests that fish indeed have the capacity to perceive pain (Brown, 2015; Douglas et al., 2018; Perry and Nevarez, 2018; Walters, 2018). Initially this was shown in trout but by now also carp, goldfish, zebrafish and a continuously growing number of other fish species have been tested accordingly and comparisons across the species provide clues about general nociceptive and pain-related mechanisms (Dunlop and Laming, 2005; Nordgreen et al., 2007; Reilly et al., 2008a; Wolkers et al., 2013; Ludvigsen et al., 2014).

The question remains, which neurological structures and circuits in zebrafish are involved in experiencing pain. Fish lack the neocortex known for processing sensory information in mammals but show increased activity in the forebrain after adverse stimuli (Reilly et al., 2008b). Similar to birds, the pallium could play an important role in pain processing (Douglas et al., 2018). Wee et al. (2019) showed convincingly that hypothalamic oxytocin neurons can be activated by Trpa1 mediated stimuli and that activation of these neurons alone is sufficient to cause nocifensive behavior. Interestingly, pain-associated emotions of stress and fear have both been located to habenula (Lee et al., 2010; Sivalingam et al., 2020). Separating the sensory from the emotional part and differentiating pain from other related emotions remains to be challenging for the localization of pain-specific structures.

As one consequence to these recent findings, there is now increased interest in developing and improving methods related to pain in fish to refine treatment with anesthetics and analgesics for all fish involved in potentially painful experiments (Readman et al., 2017; Chatigny et al., 2018; Martins et al., 2019). Second, there are still many unresolved questions about how pain is perceived and how it affects fish behavior, so further basic research in this area is urgently needed. Zebrafish models are especially well suited to tackle some of these questions as they allow a combination of non-invasive imaging of brain activity and observation of behavior that is unique in major model organisms (Leung et al., 2013; Dunn et al., 2016).

With a wide variety of tools already available for zebrafish research, these methods can now be applied to the field of pain research to advance discoveries in areas of basic pain mechanisms, neuronal circuits involved in pain, evolution of pain in comparison across species or drug screenings to discover new therapeutic targets and substances that can alter pain perception (Curtright et al., 2015; Bosse and Peterson, 2017; Bedell et al., 2018; Lin et al., 2018; Yang et al., 2018; Ko et al., 2019).

This review aims to illustrate the most important methods that have been used to investigate pain and nociception in zebrafish and to discuss how emerging techniques can contribute to the understanding of pain.

## Models of Acute and Ongoing Pain

### Stimuli and Modalities

Activating nociceptors with chemical or pharmacological means is the first choice for most research questions for acute and ongoing pain in fish. Low molecular weight substances are usually stable for a long time and can easily be tested in varying doses just by addition to the water or for adult fish via injection. Here, one has also to give the route of application some thought to fit the desired model of pain. While immersing zebrafish in algogenic substances is often preferred, injection can be superior when carefully applied. Usually it is preferable to avoid injections due to additional stress for the fish caused by handling and anesthetization before injection. However, injections offer better control for effective concentration in the fish, surpassing mechanism of bio-modification and can model localized pain effects or treatments.

A number of substances have been described that can activate nociceptors via changes of pH or as binding agonist. The administration of acetic acid as a pain-causing algogen is well established in different fish species including zebrafish (Reilly et al., 2008a; Costa et al., 2019; Soares et al., 2019). In addition, application of hypertonic NaCl solution to adult zebrafish cornea can induce nociceptive effects, which were reversed by addition of Trpv1 antagonist capsazepine (Magalhaes et al., 2018; Soares et al., 2019). Allyl isothiocyanate (AITC) from mustard oil or cinnamaldehyde act as agonist of Trpa1a and Trpa1b and can model thermal hyperalgesia (Prober et al., 2008; Curtright et al., 2015; Ko et al., 2019). Table 1 summarizes substances with algogenic potential that were applied on more than one occasion in zebrafish.

**TABLE 1:** Low molecular weight substances with algogenic potential.

Substance	Target	Application in Larvae (L) or Adults (A)	Quote
Acetic acid	Trpv1 ASIC	(L) immersed in 0.0025–0.025% (A) injected 10 μl intraperitoneal with 2.5–5% (A) injected 5 μl in lips with 0.1–10%	Steenbergen and Bardine, 2014 Costa et al., 2019 Reilly et al., 2008a; Taylor et al., 2017; do Nascimento et al., 2018; Soares et al., 2019
AITC	Trpa1a/b	(L) immersed in 0.5–100 μM (A) injected 5 μl in lips with 10 μM	Caron et al., 2008; Prober et al., 2008; Gau et al., 2013; Kokel et al., 2013; Curtright et al., 2015; Ko et al., 2019; Wee et al., 2019 Esancy et al., 2018
Capsaicin	Trpv1	(L) **no effect** for immersion in up to 300 μM (A) injected 5 μl in lips with 40.93 μM	Gau et al., 2013 do Nascimento et al., 2018; Soares et al., 2019
Cinnamaldehyde	Trpa1a/b	(L) immersed in 500 μM (A) injected 5 μl in lips with 0.33 μM or 40 mM	Prober et al., 2008 Taylor et al., 2017; do Nascimento et al., 2018; Soares et al., 2019
Formalin	Trpa1	(A) injected 5 μl in lips or tail with 0.1%	Magalhaes et al., 2017; do Nascimento et al., 2018; Soares et al., 2019
NaCl	Trpv1	(A) cornea treated with 5 M	Magalhaes et al., 2018; Soares et al., 2019
Optovin	Trpa1b	(L) immersed in 10 or 25 μM (A) immersed in 50 μM	Kokel et al., 2013; Wee et al., 2019 Kokel et al., 2013

**The algogenic potential of these substances is attributed to activation of members of the transient receptor potential (TRP) channel family or acid-sensing ion channels (ASIC). Whether formalin acts via Trpa1a or Trpa1b has not been determined in more detail.**

Capsaicin, as a well-established standard test substance for rodent experiments, has shown mixed results in fish. While nociceptive behavior in adult zebrafish was reported, larvae behavior and *in vitro* activity of Trpv1 remained unchanged after application, likely because zebrafish Trpv1 lacks the binding motif similar to rabbit or chicken (Gau et al., 2013; Graham et al., 2013; Chen et al., 2016; do Nascimento et al., 2018; Soares et al., 2019). The reason for this inconsistency remains unclear. Possibly the *in vitro* system lacked accessory protein machinery for Trpv1 activation or capsaicin was able to act via other mechanisms and pathways in the adult organism. In contrast, optovin has been shown to be a specific agonist for a single TRP channel, Trpa1b (Kokel et al., 2013). In addition, optovin has been discovered in a zebrafish screen for photosensitive substances and is binding reversible to Trpa1b under UV light only (Lam et al., 2017).

Technically more challenging is a precise and timed application of heat or cold. Of this, it is easier to test overall temperature effects on larvae by either transferring them between water baths (Malafoglia et al., 2014) or to exchange water via flow-through to the desired condition (Lopez -Luna et al., 2017). Use of a thermal laser allows for precise targeting of the region of interest on the zebrafish as well as a highly defined timing of the application (Madelaine et al., 2017; Haesemeyer et al., 2018). However, it is very low throughput, difficult to determine the exact temperature generated by the laser and does not allow for cooling. A good alternative at least for larvae is the use of a dual state heating/cooling plate with small volumes of water in direct contact with the plate (Gau et al., 2013; Curtright et al., 2015).

**FIGURE 1:**
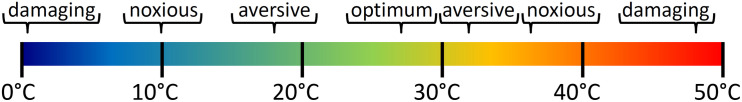
Effect of temperature on wellbeing of zebrafish larvae. Dependent on age and exposure time of larvae or adults temperatures outside the optimal range of around 23–30°C became increasingly aversive, noxious and damaging with rising heat or cold. Temperatures of 10 or 34°C have been described as noxious and are damaging at 0 or 48°C within seconds.

An overview of temperature ranges used in experiments is provided in Figure 1. Hot temperature above 30°C is aversive to larvae and 34°C is considered the threshold to noxious heat (Prober et al., 2008; Haesemeyer et al., 2018). In similar experiments, rapid cooling below 16°C was also described as noxious (Prober et al., 2008; Gau et al., 2013). Effects of cold have also been studied in the context of anesthesia and euthanasia where gradual cooling to 10–12°C caused anesthesia and rapid cooling below that point was lethal (Collymore et al., 2014; Oskay et al., 2018; Wallace et al., 2018). In contrast to larvae, adult zebrafish can slowly adapt to a wide range of temperatures but when kept at standard conditions of around 28°C similar ranges for quick changes to aversive or noxious temperatures apply as for larvae (Lopez-Olmeda and Sanchez-Vazquez, 2011).

Combining temperature aversion assays with algogenic substances can lead to further sensitization as was shown by the application of 0.5 μM AITC (Curtright et al., 2015).

To the best of our knowledge, stimuli of mechanical pressure to elicit nociception or pain without wounding have not been tested in zebrafish. Comparable experiments in goldfish and trout showed that the animals had to be restrained for a pin-prick stimulus thereby limiting the readouts to non-behavioral methods and possibly also interfering with the results due to additional stress (Dunlop and Laming, 2005).

Electricity is another classic stimulus in pain research. Here as mild classified electric shocks of 3–5 V conditioned adults for active avoidance or reduced the swimming activity of larvae (Pradel et al., 1999; Steenbergen, 2018). Larvae activity was restored when they were pre-treated with analgesic buprenorphine, indicating that indeed pain caused the change of behavior (Steenbergen, 2018). However, electric shocks represent rather unspecific stimulations that do not easily translate to a modality that zebrafish perceive in their natural environment.

Another option available in zebrafish is the direct manipulation of neurons via genetic tools. Various genetically modified lines exist that allow for ablation or to switch cells on and off with optogenetic (light), thermogenetic (temperature) or pharmacological tools, supplanting the need for more invasive and less specific methods like stimulating microelectrodes (Carr and Zachariou, 2014; Chen et al., 2016; Ermakova et al., 2017). These molecular switches can be expressed pan-neuronally but could be expressed more specifically, e.g., in nociceptors only.

Of note, there are a number of unpleasant stimuli that may trigger behavioral responses that can easily be mistaken for pain. In order to clearly detect pain specific brain activity patterns it will be crucial to test such different control stimuli. These stimuli should include salient and unpleasant stimuli that are not expected to induce nociception. This could be a fear inducing alarm substance or tapping the dish to induce stress (Wee et al., 2019; Sivalingam et al., 2020). By comparing the brain activity triggered by non-pain stimuli with that caused by pain-stimuli, clarity can be gained about the pain-specific structures and activities of the brain.

### Models of Ongoing Pain

Various models for pain have been put forward in zebrafish but so far, no single indicator is sufficient alone for concluding that fish perceive pain. The above-mentioned modalities can be used to model different types of pain, depending on the intensity, timing or the location of the applied stimulus. For example, an intraperitoneal injection of 2.5% acetic acid was used as a model for visceral pain but when injected in the lips it was used as inducer of orofacial nociception at a concentration of 0.1% and with 5% to observe the behavior in response to noxious stimuli (Reilly et al., 2008a; do Nascimento et al., 2018; Costa et al., 2019). This demonstrates modalities have to be carefully chosen as for most substances it is not yet known whether it might be noxious at a given concentration or location.

There are many animal models placing a moderate or severe burden on the fish and most of them have not been investigated regarding their pain causing potential (Xie et al., 2020). Be it toxicity tests, disease models, wounding via cuts, burns or chemicals or even acoustic trauma, with increasing damage pain is getting more likely (d’Alencon et al., 2010; Otten and Abdelilah-Seyfried, 2013; Dang et al., 2017; Huemer et al., 2017; Uribe et al., 2018). The usefulness of these models to study pain in zebrafish has yet to be proven as strong secondary effects like inflammation, chronification and habituation complicate the interpretation of results (Steenbergen, 2018; McDiarmid et al., 2019). Nevertheless, such models would be useful to investigate inflammatory or neuropathic pain for example by axon-degeneration or wound repair and regeneration after notochord injury (Malafoglia et al., 2014). Therefore, established models of other fields should be tested for potential applications in pain research.

In summary, it can be said that a wide range of different stimuli are available to induce nociception and pain in zebrafish. To achieve the desired effect, the timing, concentration and form of application have to be carefully considered though. In addition, there is still a need for models to study specific types of pain like inflammatory or neuropathic pain and already established models of other fields could be applied to pain research to advance this cause.

## Investigating Mechanisms of Nociception and Pain

Ideally, experiments should be designed in a way that multiple dimensions of the pain experience can be measured, including reflexive responses, sensory inputs and the impact on wellbeing of the zebrafish (Gregory et al., 2013). This can be achieved by a combination of the following readouts.

### Molecular Markers and Signaling

So far, only few molecular markers involved in pain pathways have been identified. The availability of zebrafish specific antibodies is rare but sometimes cross-reacting antibodies can be used like rabbit anti-oxytocin for immunohistochemistry (Wee et al., 2019). More often cellular components involved in nociception are detected on mRNA level either via quantitative PCR (qPCR) or with *in situ* hybridization (ISH) staining. These methods have been used to show the presence or absence of P2rx3b receptor or TRP-channels in tissues of interest at various timepoints (Caron et al., 2008; Prober et al., 2008; Gau et al., 2013; Graham et al., 2013).

Down-stream signaling molecule activity or changes of target gene expression have rarely been investigated. Until now, there have been only few attempts using cellular expression markers to identify states of pain or active nociception in zebrafish.

In a search for marker genes in the context of pain *c-fos*, *c-jun*, *vip*, *pacap1b*, and *bdnf* have been evaluated in zebrafish larvae (Carr and Zachariou, 2014; Malafoglia et al., 2014). After heat shock, the expression change was investigated via qPCR and *in situ* hybridization and showed strong increase for *c-fos* and *c-jun* after 30 min and for *vip*, *pacap1b* and *bdnf* after 4 h.

Prostaglandin-endoperoxide synthase 2 (PTGS2 also known as COX-2) is a known expression marker for tissue damage, chronic pain and a target for NSAIDs (Grosser et al., 2002; Ma et al., 2012). Twofold *ptgs2a* mRNA expression was detected via qPCR 30 min after exposure to diluted acetic acid in zebrafish larvae (Steenbergen and Bardine, 2014). Unfortunately, *ptgs2b*, which has even slightly better protein sequence homology with human PTGS2, has not been tested. Other known biomarkers like substance P have been identified in zebrafish but their role in pain have not been evaluated (Lopez-Bellido et al., 2013).

Staining and quantification of molecular markers may fall short to identify processes of acute pain but are better equipped to follow long-term changes of ongoing pain and secondary processes like habituation that have currently not been investigated in zebrafish.

### Electrophysiology

Much of our understanding of brain functions came from application of electrophysiological methods. These techniques allow for manipulation and measurement of voltage and current with milliseconds and millivolt precision (Dunlop and Laming, 2005; Rubaiy, 2017). Different forms have been applied in zebrafish as this technique has a great scale of application ranging from single ion channels, to cells and tissues in *ex vivo* or whole organisms (Kyriakatos et al., 2011; Baraban, 2013; Johnston et al., 2014; Roy and Ali, 2014; Eimon et al., 2018; Tsata et al., 2019).

Most of the first findings demonstrating functional nociception in teleost fish were based on extracellular recordings of neuronal activity in restrained trout and goldfish (Sneddon, 2003; Dunlop and Laming, 2005). Similar techniques have been applied in cell culture to characterize properties of zebrafish Trpv1, Trpa1a, and Trpa1b-channels (Prober et al., 2008; Graham et al., 2013; Oda et al., 2016). Whole-cell patch clamp recordings were taken on whole zebrafish larvae with exposed spinal cord to test for effects on neuronal cell ablation (Chen et al., 2016). In addition, non-invasive methods are available that use either surface-electro recordings of immobilized larvae or measurements or electric field potentials of free behaving larvae (Issa et al., 2011; Hong et al., 2016). In goldfish, patch clamp technique was applied to the large Mauthner cell that is responsible for the fast escape reflex and the effectiveness of four different anesthetics was shown (Machnik et al., 2018). Even though general function and anatomy of the Mauthner cells are also well characterized in zebrafish, these specific effects on this neuron remain to be replicated.

### Fluorescent Imaging

As a genetic model organism, the zebrafish offers a wide variety of molecular tools to study nociception, pain and associated behavior. It was estimated that a larval zebrafish brain contains 80,000–100,000 neurons (Naumann et al., 2016; Abbott, 2020). That is still small and compact enough to allow for whole brain imaging at high cellular and temporal resolution (Ahrens et al., 2013). This opens up the opportunity to use zebrafish as a model to understand animal behavior on a cellular level by using a wide variety of non-invasive imaging methods.

Numerous transgenic lines exist to label specific classes of neurons enabling the study of neuronal architecture and function. These include lines that have transgene expression in nearly all neurons or are specific to a certain neurotransmitter, cell type or tissue like sensory neurons or nociceptors (Satou et al., 2013; Marquart et al., 2015; DeMarco et al., 2019).

To establish a model of neuropathic pain 5 days post fertilization (dpf) transgenic larvae (*nsf*:Gal4/UAS:GFP)^tpl006Gt^, expressing green fluorescent protein (GFP) in a large subset of nerve cells, have been exposed for 5 s to 45°C hot water (Malafoglia et al., 2014). The transgenic line allowed the observation of nerve degeneration in combination with severely reduced swimming ability as well as regeneration starting 24 h later and regaining the ability to swim normally 48 h later.

**FIGURE 2:**
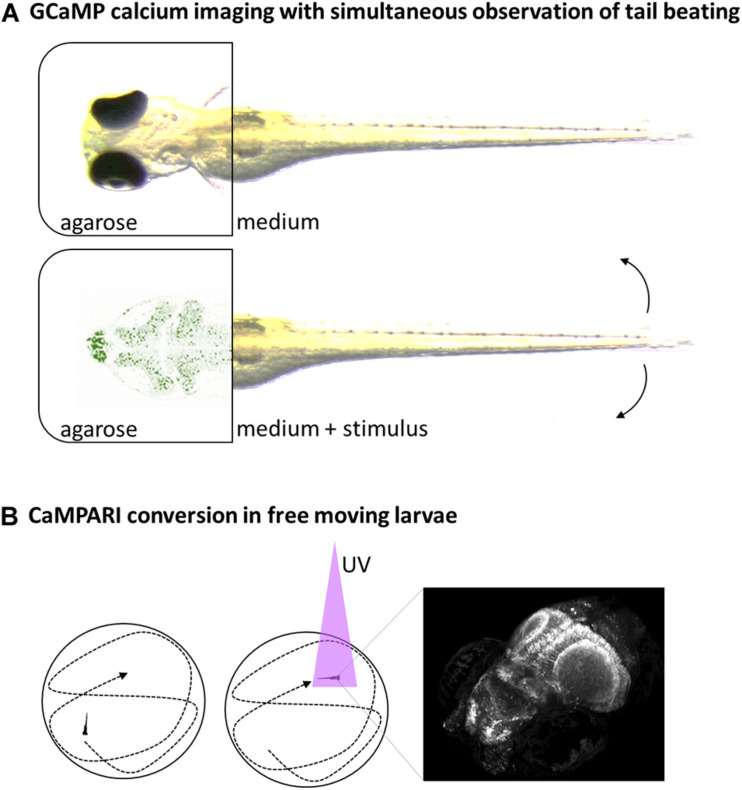
Schematic overview of selected neuroimaging methods. **(A)** Head-fixed larvae can be analyzed for neuronal activity via fluorescent Calcium-reporters like GCaMP expressed in the brain and simultaneous observation of tail beats in bright field movies. **(B)** Neuronal-specific expressed CaMPARI can be used to take a snapshot of current brain-activity in free-swimming larvae by a short burst of UV-light that facilitate conversion of CaMPARI in the presence of calcium only (left and middle). The CaMPARI converted from green to red can be imaged afterward in high resolution, revealing the neuronal activity pattern at a defined timepoint (right).

Neuronal promoters, in combination with fluorescent calcium-indicators, can offer insight in the neuronal activity of the zebrafish under different conditions. GCaMP has been one of the most popular constructs so far. It was derived from fusing calcium-binding calmodulin and GFP and strongly increases in fluorescence when calcium is bound (Nakai et al., 2001). When used in combination with a pan-neuronal promoter like *elavl3*, it acts as indicator for neuronal activity because of increased intracellular calcium concentration during synaptic potentials (Figure 2A).

Studying brain activity in this way has helped to establish the neural circuits for an increasing number of behaviors. Naumann et al. (2016) showed how distributed neurons collaborate to generate behavior and illustrate how whole-brain scans can be used to establish functional circuit models. Similarly, when Portugues et al. (2014) imaged the whole larval brain with cellular resolution they found a distributed network with an elaborate organization, where activity patterns fell into distinct clusters reflecting sensory and motor processing. When comparing data from multiple fish, a highly stereotyped pattern emerged. Combining data from multiple whole brain scans gave an average activity map.

The major challenge in these approaches is not anymore collecting but analyzing increasing amounts of data. To compare activity patterns in complex neuronal networks demands powerful computer processing capacity (Freeman et al., 2014).

An early application of this technique to study pain in zebrafish showed that when transiently expressing GCaMP in 3 dpf old larvae, increased activity could be observed in trigeminal neurons in response to a heat ramp (Gau et al., 2013). Whereas no activity was detected from 23 to 28°C, intensity and numbers of responding neurons increased with temperatures rising from 28 to 36°C. This suggests that Trpv1-expressing neurons are sensitive to noxious temperature changes that deviate from normal.

GCaMP allows studying changes in neuronal activity but powerful microscopes are needed to image a whole larvae brain in few seconds at good resolution. The calcium-sensor CaMPARI allows for decoupling of stimulation and analysis by providing a snapshot of the current active or inactive state of neuronal cells (Esancy et al., 2018). CaMPARI is similar to GCaMP but this fluorescent protein is photoconvertible from green to red fluorescence under UV-light only in the presence of calcium (Figure 2B). This feature allows labeling the current state of neuronal activity with a short burst of UV-light and then imaging the labeling slowly in high-resolution afterward. Using an *elavl3*-promoter for brain-wide expression, differences in neuronal activity were shown in free-swimming larvae under influence of anesthetics, noxious heat (45°C) or cold (4°C) to identify brain regions involved in the signaling (Fosque et al., 2015).

By successfully combining these whole-brain imaging techniques with simultaneous behavioral observations neural circuits and activity patterns for various behaviors and neural processes like heat-sensing and decision-making for movement were identified (Haesemeyer et al., 2015, 2018; Bahl and Engert, 2020; Dragomir et al., 2020; Lin et al., 2020). Noxious stimuli that signal via Trpa1 receptors were shown to activate oxytocin-positive neurons resulting in evasive behavior (Wee et al., 2019).

This demonstrates that these are powerful tools to elucidate neural processes of pain or nociception on cellular level and are able to identify the brain regions and neural circuits involved. At least for zebrafish larvae direct relations between stimulus, neural activity and behavior can be investigated. It remains to be shown how well these findings translate to adult zebrafish or other fish species.

For now calcium-imaging remains the gold-standard for neuronal mapping but there are alternatives available like the voltage indicator Bongwoori or Di-4-ANEPPS staining (Kibat et al., 2016; Okumura et al., 2018). Both methods translate electrical activity to a fluorescent response and combine brain-wide imaging with advantages of electrophysiology.

In summary, this shows that fluorescent imaging of neural processes related to nociception and pain is a new and rapidly emerging field with the potential to complement other methods of pain research in a unique way. Although still limited to larvae, there is unprecedented potential to investigate underlying mechanism of pain that might lead to discovery of pain-specific circuits in the future.

### Optogenetics

Another approach to elucidate neural circuits related to pain and nociception involves direct manipulation of nerve cells to study their function in a whole organism. One option is to target single cells with laser ablation and to observe the effects on the network (Vladimirov et al., 2018; Wee et al., 2019). More elegantly cells can be switched on and off by use of optogenetic tools (Carr and Zachariou, 2014). For example, expression of red shifted channelrhodopsin C1V1 was used to investigate the role of hypothalamic neurons in a neural circuit of nociception (Madelaine et al., 2017). After applying a nociceptive stimulus with a thermal laser to head fixed larvae, escape-like tail movements increased while activity in RFamide neuropeptide VF (NPVF) expressing neurons decreased. By expressing C1V1 in these cells, it was shown that NPVF neurons are in interdependency with neurons of the ventral raphe nucleus (vRN): Active NPVF neurons were able to suppress vRN neurons as well as the other way around when activated as part of nociceptive signals.

The recently discovered photoactive compound optovin offers similar options without the need for transgenic lines (Kokel et al., 2013). This Trpa1b-specific ligand allows for UV-light controlled activation of the channel comparable to stimulations with other known Trpa1b activators like mustard oil but with a much better control over stimulus timing (Wee et al., 2019).

### Nocifensive Behavior

Along with its popularity as a model organism, also the number of well-described and defined behaviors in both larval and adult zebrafish have been constantly growing over the last years (Kalueff et al., 2013). Pain is considered a complex emotion with many elements involved. Observing behavior and reactions of the whole body is still the gold standard to study pain in all its complexities. Recent advances in video recording, tracking and software analysis have significantly increased the throughput both for larval and adult fish (Perez-Escudero et al., 2014; Nema et al., 2016; Bai et al., 2018; Franco-Restrepo et al., 2019). Locomotion of larvae have been analyzed in 96 well plates with one zebrafish per well or up to ten unmarked adults per tank could be individually tracked and individuals were identified in a mixed group for depression and hypoactivity (Bai et al., 2018; Steenbergen, 2018).

The basic equipment needed is rather cheap compared to other methods, making it an attractive and non-invasive option when getting started with pain research. Of the large body of well described behavioral tests available for zebrafish, many still need to be evaluated for their usefulness in pain research (Malafoglia et al., 2013).

**FIGURE 3:**
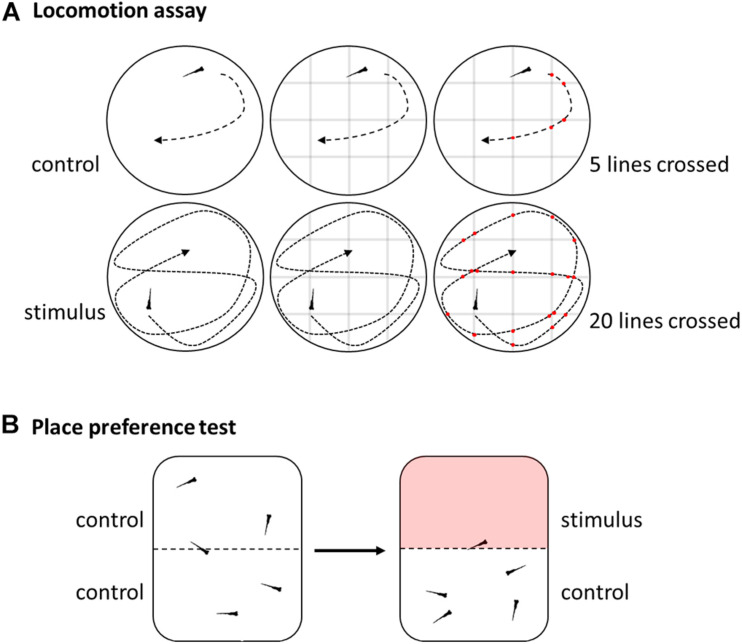
Schematic overview of selected behavioral methods. **(A)** Locomotion assay used to assess swimming activity by video tracking and either software analysis or by manually counting numbers of lines crossed of a grid digitally overlaid with the movie. **(B)** Place preference test by video analysis of reactions to aversive or noxious stimuli like heat in a dual heating plate.

A rough distinction in reflexive and non-reflexive tests can be made of the behavioral tests already employed in pain and nociception research (Gregory et al., 2013; Graham, 2016). Reflexive behavior of swimming activity/locomotion is easy to assess for both larvae and adult zebrafish in its various forms like swimming velocity or distance traveled (Figure 3A; Prober et al., 2008; Reilly et al., 2008a; Magalhaes et al., 2017; Taylor et al., 2017; do Nascimento et al., 2018; Magalhaes et al., 2018; Steenbergen, 2018; Soares et al., 2019). In addition, freezing behavior can be observed in adults or a ratio of activity and rest can be calculated (Lopez -Luna et al., 2017; Costa et al., 2019). Other good examples for reflex based behavior are models that depend on the site of injection like the abdominal constriction writhing, rubbing the lips or rubbing against objects (Reilly et al., 2008a; Costa et al., 2019).

To better distinguish emotional states of pain from other related reactions like stress, fear and anxiety more complex setups can be employed for non-reflex based tests. Most of these offer some kind of free choice allowing for insights in the decision-making. This is especially important for stimuli that could be aversive but not necessarily painful like moderately high temperatures.

The place aversion test measures the amount of time an animal spends in a certain area that differs in temperature or where chemicals have been added to the water compared to the control area (Figure 3B; Gau et al., 2013; Curtright et al., 2015). Similar, in a place preference test the animal can seek out more often or longer an area where analgesics are available (Bosse and Peterson, 2017). This approach can be further refined by involving an additional cost to be paid for accessing analgesics, giving an indication on the importance of a decision (Bosse and Peterson, 2017). In addition, some settings might require additional training or conditioning before the actual test to reduce aversion, stress and anxiety (Bosse and Peterson, 2017).

It is recommendable also to test directly for anxiety and fear in these settings, in case a chosen treatment influences these pain-related emotions, too. Here again many models have already been described (Kalueff et al., 2016; Perathoner et al., 2016; Kysil et al., 2017). In the light-dark test, a fearful zebrafish would stay rather in a dark area while bolder animals would venture outside the shadow more often (Magalhaes et al., 2017, 2018). Similar in the bottom-tank test an anxious zebrafish prefers the deep water and would rarely swim to the surface (Costa et al., 2019). In addition, anti-anxiolytic substances like diazepam were used as control (Magalhaes et al., 2017).

Taken together, this demonstrates that despite all difficulties behavioral analysis was and still is one of the main approaches to study nociception and pain in zebrafish and to assess noxious or aversive stimuli. Its advantages will be further enhanced, with the increasing feasibility of behavioral high-throughput experiments. However, observation of swimming activity alone is insufficient to infer to states of pain as both an increase and a decrease have been described following algogenic stimuli and even though the results are reproducible, they are purely descriptive and no unifying theory is available for hypothesis based testing (Taylor et al., 2017; Soares et al., 2019). It can only be speculated that the reason lies in the combination of the type of pain (nociceptive, inflammatory or neuropathic) and the amount of pain caused, as in one instant the highest concentration in a dose curved failed to elicit a significant change in activity (Taylor et al., 2017). Therefore, only combinations of various established tests are suitable to investigate different aspects of pain and to capture its full complexity.

## Analgesics

Apart from a more thorough understanding of basic mechanisms, the overall aim in zebrafish pain research has always been either to refine painful zebrafish procedures in experiments according to 3R guidelines or to discover new opportunities in translational research. Even though it is still difficult to show painful states in zebrafish, the growing number of classic analgesics that were able to restore normal behavior after nociceptive or potentially painful treatments shows impressively the parallels between the nociceptive system of fishes and mammals. These analgesic substances can be classified based on their mechanism of action as opioids, anesthetics or non-steroidal anti-inflammatory drugs (NSAIDs). An overview of selected analgesics and their effective dose in larvae (L) or adults (A) is shown in Table 2. Application of analgesics should always be considered for potentially painful procedures to remain in accordance with 3R guidelines. An optimal analgesic regimen is not only essential for animal welfare but also prevents that remaining pain interferes with the scientific outcome. The effectiveness of a given analgesic can be monitored via observation of behavior (Deakin et al., 2019). However, it should be kept in mind that the choice of analgesic can affect the scientific objective. Indeed, the use of NSAID will not only suppress pain but also inflammatory processes. This could impact the outcome when studying tissue-regeneration of the fin, heart or nervous system. Similarly, opioids and anesthetics can have systemic effects and act on the central nervous system (Bao et al., 2019; de Abreu et al., 2019). This should be noted when aiming for behavioral outcomes, but side effects can be avoided when dose range is carefully chosen (Deakin et al., 2019). In addition, Lelek et al. (2020) showed recently how they selected an appropriate analgesic for heart regeneration after cryoinjury. This can be seen as a general roadmap for other models too, how pain can be alleviated while avoiding side effects.

**TABLE 2:** Selected analgesics and their effective doses in larvae (L) and adult (A) zebrafish.

Substance	Class	Application in Larvae (L) or Adult (A)	Quote
Morphine	Opioid	(L) immersed in 48 mg/l (A) injected 5–20 μl IP with 200 mg/l (A) injected IP with 8 mg/kg (A) injected IM with 2.5 and 5 mg/kg (A) injected IP with 5 mg/kg	Lopez -Luna et al., 2017 Magalhaes et al., 2017 Magalhaes et al., 2018 Taylor et al., 2017 Costa et al., 2019
Buprenorphine	Opioid	(L) immersed in 5 μM (L) immersed in 0.1 mg/l	Curtright et al., 2015 Steenbergen and Bardine, 2014
Lidocaine	Anesthetic	(L) immersed in 5 mg/l (A) immersed in 300 mg/l (A) injected IM 1 mg/kg (A) immersed in 2 and 5 mg/l	Lopez -Luna et al., 2017 Collymore et al., 2014 Sneddon, 2012 Schroeder and Sneddon, 2017; Deakin et al., 2019
Acetylsalicylic acid	NSAID	(A) immersed in 1 and 2.5 mg/l	Schroeder and Sneddon, 2017
Diclofenac	NSAID	(A) injected IP with 40 mg/kg	Costa et al., 2019
Indomethacin	NSAID	(A) injected 5–20 μl IP with 200 mg/l	Magalhaes et al., 2017

**Analgesics were applied either via immersion, injection intraperitoneal (IP) or injection intramuscularly (IM).**

### Opioids

Analgesic effects of various opioids have been successfully demonstrated in both larvae and adult zebrafish. The mechanisms of the opioid system have been well investigated and extensively reviewed elsewhere (Gonzalez-Nunez and Rodriguez, 2009; Demin et al., 2018; Bao et al., 2019). In brief, it has been shown that zebrafish have several opioid receptor homologs and these are responsible for mediating opioid effects on the central nervous system, e.g., normalizing behavior after nociceptive stimuli but also adverse effects like respiratory depression or withdrawal effects after conditioning (Bosse and Peterson, 2017; Zaig et al., 2020). In addition, the competitive antagonist naloxone has been shown to block the effects of opioids in a concentration dependent manner (Steenbergen and Bardine, 2014; Taylor et al., 2017).

### Anesthetics

In contrast to opioids that act on an endogenous system to modulate pain perception, anesthetics block nerve conduction like nociceptive transmissions in a treated area altogether by inhibiting voltage-gated sodium channels.

General anesthesia is easily achieved by immersing zebrafish in anesthetic solutions. Affecting the central nerve system and depending on the concentration the result is sedation or unconsciousness. While tricaine (MS-222) is often the preferred drug for general anesthesia, it has proven to be too ineffective in injections for local anesthesia, as it is quickly eliminated (Carter et al., 2011). Instead, lidocaine has shown promising results in inducing analgesia via immersion and combinations with other substances might proof beneficial (Huang et al., 2010; Collymore et al., 2014; Valentim et al., 2016; Deakin et al., 2019). Even though lidocaine did not influence behavior of adult zebrafish at 5 mg/l, it impaired heart regeneration showing the need to carefully control for side effects in any given procedure (Deakin et al., 2019; Lelek et al., 2020).

### NSAIDs

The main analgesic effect of NSAIDs is mediated via blocking prostaglandin synthesis by inhibiting cyclooxygenase enzyme COX-1 or COX-2. However, NSAIDs like acetylsalicylic acid and ibuprofen failed to restore normal behavior in acute and sensitized larval temperature avoidance assays (Curtright et al., 2015; Lopez -Luna et al., 2017). In contrast, in adults, acetylsalicylic acid was beneficial after tail fin clip (Schroeder and Sneddon, 2017) and indomethacin after formalin injection (Magalhaes et al., 2017). Interestingly indomethacin was as effective in the first 5 min considered before the onset of the inflammatory phase as well as in later stages. With regard to side effects it was shown that inhibition of COX enzymes by NSAIDs have caused gastrointestinal damage by disrupting the epithelial layer, thereby actually inducing inflammation (Goldsmith et al., 2013). Therefore, a more systematic and comprehensive testing is needed to identify mechanisms how NSAIDs act in zebrafish and if for example they are only beneficial in inflammatory pain or only in adults but not in larvae.

## Replace, Reduce, Refine

Current animal welfare concepts apply to all animal experiments. This will often cause considerable challenges for the study of pain. Even if the topic of pain in fish is not conclusively clarified, pain, suffering and harm must be minimized. In accordance with the 3Rs concept of Russell and Birch (Russell and Burch, 1959), alternative methods have to be used where possible, the number of animals required must be reduced and the experimental and husbandry conditions have to be improved. Although there is no full consensus regarding the best housing conditions converging evidence suggests that simple enrichment measures can be easily applied for improving animal welfare (Stevens et al., 2021). With regard to alternative methods, at least formally, the use of pre-feeding fish can be considered as a means for replacement (Sloman et al., 2019). This is due to current legislation, which for example in the EU consider pre-feeding fish not as sentient. However, we would like to make it clear that alternative methods without the use of live animals have to be used wherever possible. In order to reduce the number of test animals, it should be noted that there are a number of useful tools for calculating suitable sample sizes (Festing, 2018). Especially in animals like *Danio rerio* that can quickly be generated in large numbers, sample sizes have to be sufficient to provide the power required for reproducible results but not larger, just as in all regulated animal experiments. In addition, surplus animals derived from breeding shall be considered in order to further reduce the number of laboratory animals related to scientific experiments (Lewejohann et al., 2020a). Refinement approaches must include the use of analgesia and anesthesia where necessary and applicable without compromising the objective of the experiment. In addition to the design of experimental conditions under the premise of the least possible impairment of well-being, special attention must be paid to the conditions outside the experiment (Lewejohann et al., 2020b). Here, there is a considerable need for research on the improvement of housing and living conditions under laboratory settings (Stevens et al., 2021).

## Conclusion and Future Directions

The last 10 years have seen a rising interest in studying basic mechanism of pain and nociception in zebrafish and an advancement of the available methods along the way. While still under discussion if the fish brain is sufficiently evolved to encode the perception of pain, an increasing number of methods allow at least investigation of the detrimental effects of noxious stimuli.

Some stimuli have been proven more reliable than others have and acetic acid, AITC and formalin treatments are now among standard applications. Analgesic properties of opioids have been confirmed while NSAIDs and especially lidocaine among local anesthetics merit further investigation. All forms of analgesia still need a more thorough investigation of possible side effects.

Measuring the awareness of pain remains challenging not only in zebrafish, but in all non-primate animal models. Change of behavior is still the gold standard as readout. Here new advances in recording, tracking and software-based analysis facilitate high-throughput approaches and transition from reflexive to non-reflexive behavior as more meaningful readout. Still, the impossibility to measure directly an emotion like pain requires combination of several methods to capture its complexity. While the effectiveness of analgesics is a good indicator, fluorescent imaging of neuronal activity is promising to decipher the behavior-underlying mechanisms. It remains to be seen if whole-brain analysis of neuronal activity and circuits offers better understanding of pain-related behavior or even a new form of biomarkers of acute pain. Indicative for that possibility is the discovery of certain neurons that seemed to signal a certain brain state, maybe even emotions, that were used to predict different reactions to otherwise identical stimuli (Abbott, 2020). Combinations of behavioral observation with neuronal imaging could be a way forward to achieve a more objective and quantifiable measurement of pain awareness.

Still missing in the toolbox for zebrafish pain research is a long-term model for ongoing or chronic pain. Obviously, these kind of animal experiments have to be well prepared and to avoid unnecessary suffering a profound knowledge of basic pain mechanisms in fish are prerequisite. Nevertheless, at the same time a better understanding for how long fish have to be treated with analgesics after procedures and how pain influences recovery and healing is necessary for improved animal welfare. Eventually pain and suffering could become more objectively quantifiable allowing for refined severity classifications of animal experiments. To date it remains a major challenge for animal welfare to detect and treat pain in fish, in day-to-day husbandry or animal experiments not focused on pain research. It remains to be seen, how increasing knowledge on nociception and pain will be transferred to daily husbandry procedures to reduce pain in all vertebrates to the unavoidable minimum as required by European law.

Even though both larvae and adult zebrafish have been applied to study nociception, further research is required how the response to noxious stimuli might change during development and which methods are best suited for a certain developmental stage. This data would also be important to gauge how well findings from larvae studies would translate to adult fish or even to humans, a prerequisite before screening efforts are wasted (Mogil, 2019; Withey et al., 2020). In addition, it has to be considered that employing zebrafish larvae in pain models equals acknowledging their capacity for pain and even though most countries do not have legal protection for these early stages of development, it would be unethical denying them improved refinement in form of analgesics or otherwise when possible (Sneddon, 2018; Kelly and Benson, 2020).

Zebrafish have been recognized as an attractive model for drug screens, allowing greater numbers in throughput compared to other vertebrate systems (Kalueff et al., 2014). Now, with pain and nociception models becoming more refined and reliable, there is a growing interest in screens for analgesics, too (Gonzalez-Nunez and Rodriguez, 2009; Demin et al., 2018; Bao et al., 2019). Monitoring brain activity in high numbers emerges as a new option to screen for effects on the nervous systems (Lin et al., 2018) and a platform to study the needs of zebrafish in form of self-administration has been introduced (Bosse and Peterson, 2017). These offer new options to search for substances to treat animal or human pain.

Models that were developed in the last years to elucidate the nociceptive pathway and mechanisms of pain in rodents can offer guidance for new applications in fish in the near future.

For the identification of pain pathways in rodents, optogenetic and chemogenetic tools are of great value (Whissell et al., 2016; Huang et al., 2018). However, whereas optogenetic tools were already used in zebrafish, chemogenetics, like designer receptors exclusively activated by designer drugs (DREADDs), could still be applied in future. Indeed, the specific inhibition or activation of certain groups of neurons, through engineered G protein-coupled receptors, could give new information about processing of nociceptive information in zebrafish.

Furthermore, in recent years in multiple research areas, the automated detection of very subtle behavioral changes advanced defining a broad range of behaviors in different organisms. The application of machine learning furthered establishing a grimace scale in mammals as an indicator for pain (Langford et al., 2010; Sotocinal et al., 2011; Andresen et al., 2020). Automated analysis systems for the walking behavior of rodents are discussed as an objective assessment of spontaneous and ongoing pain behavior in rodents (Vrinten and Hamers, 2003; Xu et al., 2019). Even in fruit flies, small changes in wing angle could be associated with the activity of specific neurons (Robie et al., 2017). In fish, the detection of a grimace scale seems rather unlikely; however, changes in fin position or general alterations in swimming gesture might help identify spontaneous pain behavior.

A very prominent difference, when comparing pain literature from rodents with pain models in fish, is the near absence of tests involving mechanical stimulation. This is certainly due to the difficulty of applying a standardized mechanical stimulus to a swimming organism. However, in restrained zebrafish, like the ones used for the imaging experiments, this seems imaginable to establish. Since biting plays an important role in social interaction in zebrafish, the investigation of mechanical nociception could provide interesting new knowledge.

In summary, we conclude that combinations of established zebrafish models offer great potential to elucidate pathways of nociception and acute pain. With this review, we provide a toolbox for other researchers to investigate effects of pain and its treatment in zebrafish to improve animal welfare and further our understanding of underlying mechanisms.

## Author Contributions

NO drafted the manuscript and prepared figures and tables. CH and LL revised the draft and provided expertise input on mouse models and refinement. All authors approved the final version.

## Conflict of Interest

The authors declare that the research was conducted in the absence of any commercial or financial relationships that could be construed as a potential conflict of interest.

## Abbreviations

3R: replace reduce refine
AITC: allyl isothiocyanate
ASIC: acid-sensing ion channel
dpf: days post fertilization
GFP: green fluorescent protein
ISH: *in situ* hybridization
NSAID: non-steroidal anti-inflammatory drug
qPCR: quantitative PCR
TRP: transient receptor potential channel.
